# Do bactisealventriculostomy catheters have a low rate of ventriculitis and catheter colonization in clinical practice?

**DOI:** 10.1186/1743-8454-7-S1-S41

**Published:** 2010-12-15

**Authors:** Roger Strachan, Himanshu Shekhar, Shuhaib Dambatta, Pratipal Kalsi

**Affiliations:** 1 Department of Neurosurgery James Cook University Hospital Marton Road Middlesbrough TS9 6JE, UK

## Background

While Bactiseal catheters have been shown to decrease ventriculo-peritoneal shunt infection rates, similar evidence for external ventricular drains (EVD) is lacking [1]. This retrospective study was undertaken to evaluate whether the introduction of Bactiseal EVD in 2004 has decreased infection rates.

## Materials and methods

One hundred and ten patients had undergone EVD insertions from January 2000 to March 2008. After excluding patients who had died within 5 days of the procedure and also those with pre-existing CSF sepsis, a total of 90 patients (with 119 EVD insertions- 66 Standard, 53 Bactiseal) were included in the study. The practice of sending catheter tips for culture was started with the introduction of Bactiseal drains. The parameters studied were age, sex, ASA score, grade of surgeon, significant medical history, trauma, concurrent surgeries, revisional surgery, peri-operative antibiotic, Bactiseal, tunnelling and duration of drainage. The incidence of external ventricular drain related ventriculitis, catheter tip colonisation and the responsible pathogens were noted.

## Results

Eleven cases of ventriculitis were recorded and the majority were caused by Gram positive bacteria. Both Bactiseal and standard catheters were associated with similar infection rates (Fishers Exact test, p= 0.53). A significantly longer average length of in-situ stay of ventricular catheters was observed in the cases developing ventriculitis (Kolmogorov-Smirnov test, p<0.01). The Bactiseal catheters took longer time to develop infection as compared to Standard catheters. But, this difference was not statistically significant (Kaplan-Meier survival analysis, p = 0.12). Analysis comparing infection incidence for the rest of the risk factors shows that only revisional surgery was associated with higher infection rates (Fishers Exact test, p= 0.03). Twenty sixBactiseal catheter tips were sent for culture, including catheter tips for 6 cases with ventriculitis. Only 2 catheters were found to be colonised and Pseudomonas was cultured from both (1 with and 1 without associated ventriculitis).

## Conclusions

Ventriculostomy related infections are strongly associated with longer in-situ stay of ventricular catheters. Bactiseal catheters may be the preferred option in those patients needing prolonged ventricular drainage. As compared with historical controls [2], Bactisealventriculostomy catheters have a low rate of colonisation and Pseudomonas seems to be the major culprit. 1. Prevention of infection in neurosurgery: role of "antimicrobial" catheters.
